# Assessment of causal associations between obesity and peripheral artery disease: a bidirectional Mendelian randomization study

**DOI:** 10.3389/fcvm.2024.1332530

**Published:** 2024-05-06

**Authors:** Xi-wei Huang, Shu-wen Pang, Tao Zhang, Chuang-wei Huang

**Affiliations:** ^1^ Department of Emergency Medicine, Puning People’s Hospital, Jieyang, Guangdong Province, China; ^2^ Department of Hygiene and Health, Guangzhou South China Business School, Guangzhou, Guangdong Province, China; ^3^ Department of Graduate Studies, Guangzhou University of Traditional Chinese Medicine, Guangzhou, Guangdong Province, China; ^4^ Department of Cardiology, General Hospital of the Southern Theater Command of the People’s Liberation Army of China, Guangzhou, Guangdong Province, China; ^5^ Department of Emergency Medicine, The Affiliated Brain Hospital of Guangzhou Medical University, Guangzhou, Guangdong Province, China

**Keywords:** Mendelian randomization, obesity, peripheral artery disease, atherosclerosis cardiovascular disease, metabolic diseases

## Abstract

**Background:**

Several observational studies have documented a potential link between obesity and peripheral artery disease (PAD), although conflicting findings exist. The causal relationship between obesity and PAD continues to be a subject of ongoing debate in the medical community.

**Objectives:**

In this study, we employed a bidirectional Mendelian randomization (MR) analysis to explore the potential causal relationship between obesity and the risk of PAD.

**Methods:**

To investigate these causal relationships, we conducted bidirectional MR analysis using publicly available genome-wide association study (GWAS) data. Effect estimates were calculated using the random-effects inverse variance-weighted (IVW) method.

**Results:**

We identified eight independent single nucleotide polymorphisms (SNPs) associated with obesity in 218,735 samples involving 16,380,465 SNPs, all of which met the genome-wide significance threshold (*p *< 5 × 10^−^⁸). The IVW analysis indicates a significant positive association between genetic obesity and multiple datasets with PAD as the outcome: Queue-1 (GWAS ID: finn-b-I9_PAD) (OR = 1.138, 95% CI: 1.027–1.261, *p *= 0.013), Queue-2 (GWAS ID: bbj-a-144) (OR = 1.190, 95% CI: 1.019–1.390, *p *= 0.028), Queue-3 (GWAS ID: ebi-a-GCST90018670) (OR = 1.174, 95% CI: 1.014–1.360, *p *= 0.032), and Queue-4 (GWAS ID: ebi-a-GCST90018890) (OR = 1.194, 95% CI: 1.099–1.296, *p *< 0.001). However, we did not observe a significant genetic-level association between obesity and PAD for Queue-5 (GWAS ID: ukb-d-I9_PAD) (OR = 1.001, 95% CI: 1.000–1.002, *p *= 0.071). Furthermore, we conducted a reverse causal MR analysis to explore the potential reverse causal relationship between obesity and PAD. This comprehensive analysis did not provide evidence of a reverse causal association between these two factors.

**Conclusions:**

In summary, our study offers genetic evidence suggesting a possible causal link between obesity and PAD. While we did not find evidence supporting the “obesity paradox”, prudent weight management remains crucial, as lower weight does not necessarily guarantee better outcomes. As with any study, caution is required in interpreting the findings. Further research is essential to assess the clinical relevance of weight in preventing PAD, which could inform the development of more precise intervention strategies.

## 1 Introduction

PAD is a vascular disorder primarily caused by atherosclerosis, defined as the partial or complete narrowing of one or more peripheral arteries (1). The prevalence of PAD ranges from 3% to 10%, affecting over 200 million people worldwide, leading to a spectrum of clinical presentations from subclinical to severely limiting lifestyles (1, 2). PAD has several classification systems based on the varying clinical manifestations in patients, with the Rutherford classification being one such system (3). Patients with no apparent clinical symptoms (but possibly with imaging evidence) are categorized as Rutherford stage 0. Those with mild clinical symptoms, such as intermittent claudication, fall into Rutherford stage 1. Those with moderate to severe claudication are classified as Rutherford stage 2. Patients experiencing rest pain are classified as Rutherford stage 3, with none of the stages from 0 to 3 involving tissue necrosis. If a patient presents with small areas of necrosis, they are categorized as Rutherford stage 4. Those with larger ulcerations and necrosis but without full foot gangrene are designated as Rutherford stage 5. Progressing to Rutherford stage 6 represents the most severe stage of PAD, characterized by full foot gangrene (3). It is worth noting that PAD not only frequently leads to severe limb adverse events such as amputation, significantly impacting patients' quality of life, but also increases the risk of cardiovascular disease and death by 3–6 times (4, 5). Through the study and management of PAD risk factors, the incidence of adverse events such as amputation can be greatly reduced (4, 5).

Obesity is a state characterized by an excess accumulation of body fat, typically resulting in a substantial deviation from the normal weight range, with obesity defined (6) as a body mass index (BMI) of ≥30 kg/m^2^. Obesity can have a significant impact on health, making individuals susceptible to various complications, including metabolic syndrome, type 2 diabetes, joint issues, specific cancers, as well as a range of cardiovascular diseases, including coronary artery disease (CAD) and potential PAD (6). Numerous studies in the past have explored the connection between obesity and PAD. A large-scale cohort study from Israel, involving 10,059 males aged 40–65, and following up with 8,343 individuals without symptoms of coronary heart disease or PAD for 5 years, found that males with higher BMI levels had a higher risk of intermittent claudication (7). Simultaneously, in recent years, an observational study from the United States, including 3,250,350 participants, with 65.5% being women, revealed that an increased BMI was a robust independent risk factor for PAD in women (8). However, some studies have yielded inconsistent results. An epidemiological study focusing on individuals aged 65 and older in Africa demonstrated a significant association between underweight (OR: 2.09, *p *= 0.0009) and obesity (OR: 1.90, *p *= 0.0336) with PAD after adjusting for potential confounding factors (9). However, in multivariate analysis, there was no significant association between overweight and PAD (9). Meanwhile, another multicenter cross-sectional study from Spain, involving 28 primary healthcare centers and a population aged >49 years, demonstrated that a BMI ≥25 kg/m^2^ (OR: 0.57, 95% CI: 0.38–0.87) and walking >7 h per week (OR: 0.67, 95% CI: 0.49–0.94) were similarly identified as protective factors against PAD (10). The connection between obesity and PAD seems to present an “obesity paradox,” likely due to the predominant reliance on observational studies for evidence. Given the inherent difficulties in fully mitigating the impact of residual confounding and reverse causality in observational research, a conclusive causal relationship between obesity and PAD remains elusive at this time.

Regarding causality, MR has become an increasingly utilized method, harnessing data from recent GWAS. Genetic variations in the phenotype can function as instrumental variables (IVs), thereby illuminating the causal relationship between exposure and outcome, while mitigating the impact of potential confounding factors often encountered in observational studies (11). A MR study involving 11,477 adults aged 40 and above from Shanghai, China, found that after controlling for potential intermediate factors such as hypertension, lipid abnormalities, and hyperglycemia, obesity may have a causal relationship with PAD (12). Another recent MR study, using genetic association summary data from consortia and the UK Biobank, comprising 140,595–898,130 individuals primarily of European descent, yielded similar results. It indicated that reducing the obesity rate could decrease the risk of cardiovascular diseases, including PAD (13). However, both of these studies relied on genetic association estimates for PAD from a single dataset. In recent years, several genetic association datasets for PAD have emerged from GWAS, allowing the opportunity for cross-validation across multiple datasets to enhance result reliability. MR is a method used to estimate causal relationships under specific assumptions. In this study, we hypothesized a positive association between obesity and PAD. We employed the MR method to explore the potential causal relationship between obesity and PAD while adjusting for traditional risk factors associated with PAD, including hypertension, lipid abnormalities, hyperglycemia, and smoking. Additionally, we conducted several supplementary analyses to assess the robustness of our results.

## 2 Materials and methods

### 2.1 Study design

**Figure 1:**
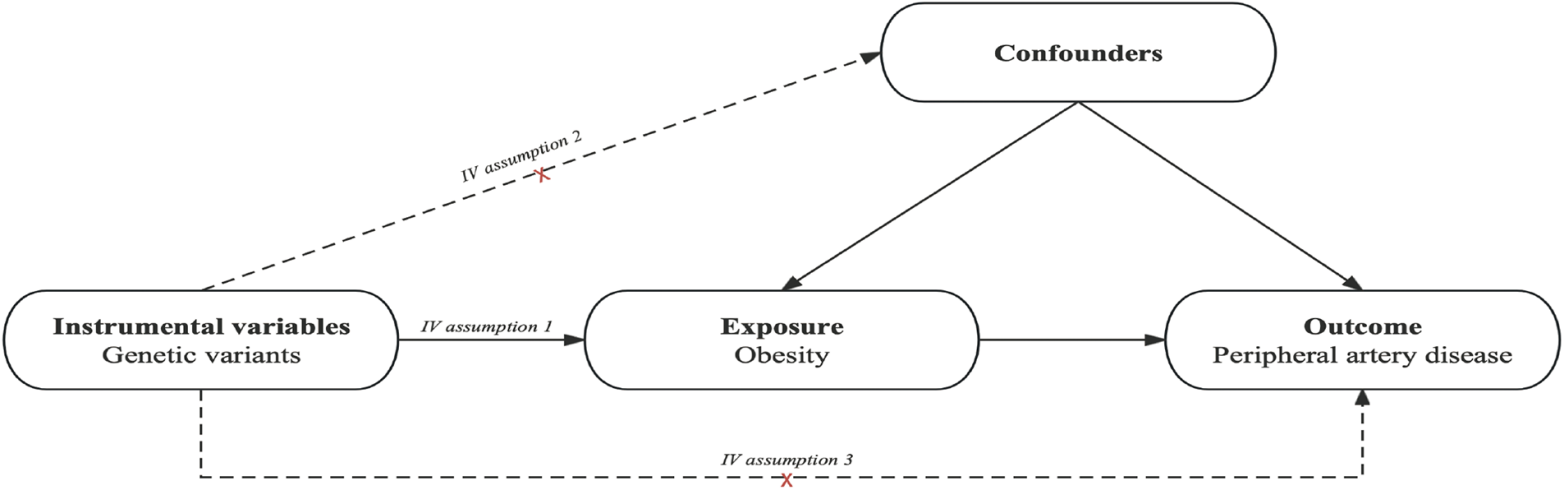
Causal diagram. The causal diagram for Mendelian randomization (MR) involves instrumental variables (IVs) and requires three assumptions: (1) single nucleotide polymorphisms (SNPs) are strongly associated with obesity; (2) SNPs are independent of known confounders; (3) SNPs only affect Peripheral artery disease (PAD) via obesity.

The schematic view of the study design and the three key assumptions of MR (11) are shown in Figure 1 and are as follows: (A) SNPs are strongly associated with obesity, (B) SNPs are independent of known confounders, and (C) SNPs only affect PAD via obesity (Figure 1).

### 2.2 Data sources

The analysis was conducted using publicly available summary-level data from GWAS studies that focused on traits of interest. These studies predominantly featured European and East Asian populations, including both males and females, and were published between 2018 and 2021. We employed the assessment methods and diagnostic criteria as published in the original studies to evaluate outcomes and other relevant variables. In our study, quantitative variables, including key indicators like BMI and cholesterol levels, were measured using their respective standard units. The genetic association estimates for obesity were obtained from GWAS databases, primarily focusing on European populations, which included 8,908 cases and 209,827 controls, spanning both males and females (14). The genetic association estimates for PAD (ukb-d-I9_PAD) were derived from the data reported by the Neale lab, predominantly involving European populations, with 1,230 cases and 359,964 controls, encompassing both males and females (15). Additionally, the genetic association estimates for PAD (finn-b-I9_PAD) were extracted from GWAS databases, primarily focused on European populations, comprising 7,098 cases and 206,541 controls, involving both males and females (16). Furthermore, the genetic association estimates for PAD (bbj-a-144) were based on findings reported by Ishigaki K, primarily involving East Asian populations, with 3,593 cases and 208,860 controls, encompassing both males and females (17). Likewise, the genetic association estimates for PAD (ebi-a-GCST90018670) were obtained from findings reported by Sakaue S, predominantly focusing on East Asian populations, including 4,112 cases and 173,601 controls, spanning both males and females (18). Lastly, the genetic association estimates for PAD (ebi-a-GCST90018890) were extracted from findings reported by Sakaue S, predominantly involving European populations, with 7,114 cases and 475,964 controls (18). The data used in this work are publicly available and the studies from which they were obtained are cited. All these studies obtained relevant participant consent and ethical approval. The results from the analyses performed in this work are presented in the main manuscript or its supplementary files. All code used for this work is available upon reasonable request to the corresponding author. This paper has been reported based on recommendations by the STROBE-MR Guidelines (Research Checklist) (19). The study protocol and details were not pre-registered.

### 2.3 Selection and validation of SNPs

Three criteria guided the selection of suitable SNPs (11). Initially, we identified SNPs associated with obesity at the genome-wide significance threshold (*p *< 5 × 10^−8^). To ensure the independence of the selected SNPs, we evaluated pairwise linkage disequilibrium (20). Any SNPs with *r*^2^*^ ^*> 0.001 (within a clumping window of 10,000 kb) were excluded if they displayed high correlation with other SNPs or had a higher p-value. Subsequently, we calculated the F-statistic to assess the strength of individual SNPs. SNPs with F-statistics greater than ten were considered sufficiently robust to mitigate potential bias. Additionally, we addressed missing data using multiple imputation methods. It's important to note that the genotype data used in this study were complete, eliminating the need for further missing data handling. Before conducting the MR analysis, data harmonization steps were taken to ensure that the effects of an SNP on the exposure and outcome corresponded to the same allele. To prevent IVs from being associated with potential confounding factors that could influence both obesity and PAD, we systematically searched for each selected SNP and its proxies in databases such as Phenoscanner (http://www.phenoscanner.medschl.cam.ac.uk/) (21) and the GWAS catalog (https://www.ebi.ac.uk/gwas/) (22). In this study, confounding factors included smoking, hypertension, diabetes, and dyslipidemia. Subsequently, we performed the MR analysis as mentioned earlier after excluding SNPs that exhibited associations with these relevant confounding factors or with PAD. This comprehensive approach ensured the reliability and robustness of our Mendelian randomization analysis.

### 2.4 MR analysis

An IVW meta-analysis was performed as the primary analysis using a random-effects model. In addition, two sensitivity analyses were conducted: the weighted median method and MR-Egger method. The weighted median method can provide reliable estimates when more than 50% of the information is derived from IVs (23). The MR-Egger method can be used to evaluate the presence of horizontal pleiotropy among the selected IVs (24).Cochrane's Q-value indicated heterogeneity among the selected IVs. Additionally, we conducted a leave-one-out sensitivity analysis to evaluate whether individual SNPs exerted a disproportionate influence on the overall estimates. All statistical analyses were conducted using the “TwoSampleMR” package in R version 4.3.1 (R Foundation for Statistical Computing, Vienna, Austria).

## 3 Results

### 3.1 SNP selection and validation

In summary, the studies we included were published between 2018 and 2021 and were primarily based on European and East Asian populations (Table 1). According to the previously mentioned selection criteria, we extracted eight independent SNPs with no linkage disequilibrium from a total of 218,735 samples for the MR analysis of their association with PAD. Table 2 provides information on SNPs significantly associated with obesity, including SNP ID codes, effect alleles, other alleles, EAF values, Beta values, and standard errors (S.E.).

**Table 1:** Details of the GWAS datasets included in this Mendelian randomization.

GWAS ID	Traits	Year	Sample size	Control	Case	Number of SNPs	Population
Exposure							
finn-b-E4_OBESITY	Obesity	2021	218,735	209,827	8,908	16,380,465	European
Outcomes							
ukb-d-I9_PAD	PAD	2018	361,194	359,964	1,230	9,637,467	European
finn-b-I9_PAD	PAD	2021	213,639	206,541	7,098	16,380,453	European
bbj-a-144	PAD	2019	212,453	208,860	3,593	8,885,805	East Asian
ebi-a-GCST90018670	PAD	2021	177,713	173,601	4,112	12,454,558	East Asian
ebi-a-GCST90018890	PAD	2021	483,078	475,964	7,114	24,186,090	European

GWAS, Genome-Wide Association Studies; PAD, peripheral artery disease.

**Table 2:** Genome-wide significant SNPs for obesity.

SNP	POS	P	SS	Beta	SE	CHR	EAE	OAE	*R* ^2^	*F*	Gene
rs12623218	632146	2.98E-11	218735	0.1511	0.0227	2	A	T	0.000203	44.31	TMEM18
rs734597	50836279	7.27E-10	218735	0.1271	0.0206	6	A	G	0.000174	38.07	RP11-228O6.2
rs71245092	76679561	5.88E-10	218735	0.1327	0.0214	7	A	G	0.000176	38.45	RP11-467H10.2
rs11030104	27684517	1.42E-12	218735	-0.1592	0.0225	11	G	A	0.000229	50.06	BDNF
rs1558902	53803574	5.23E-42	218735	0.2279	0.0168	16	A	T	0.000841	184.02	FTO
rs117280037	5937705	2.46E-08	218735	0.3779	0.0678	19	C	T	0.000142	31.07	RANBP3
rs4072287	18475675	2.27E-08	218735	0.0950	0.0170	19	A	C	0.000143	31.23	PGPEP1

SNP, single nucleotide polymorphism; POS, pos. exposure; P, pval. exposure; SS, samplesize. exposure; Beta, beta. exposure; SE, se. exposure; CHR, chr. exposure; EAE, effect_allele. exposure; OAE, other_allele. exposure; F, F statistic.

### 3.2 Peripheral artery disease

**Figure 2:**
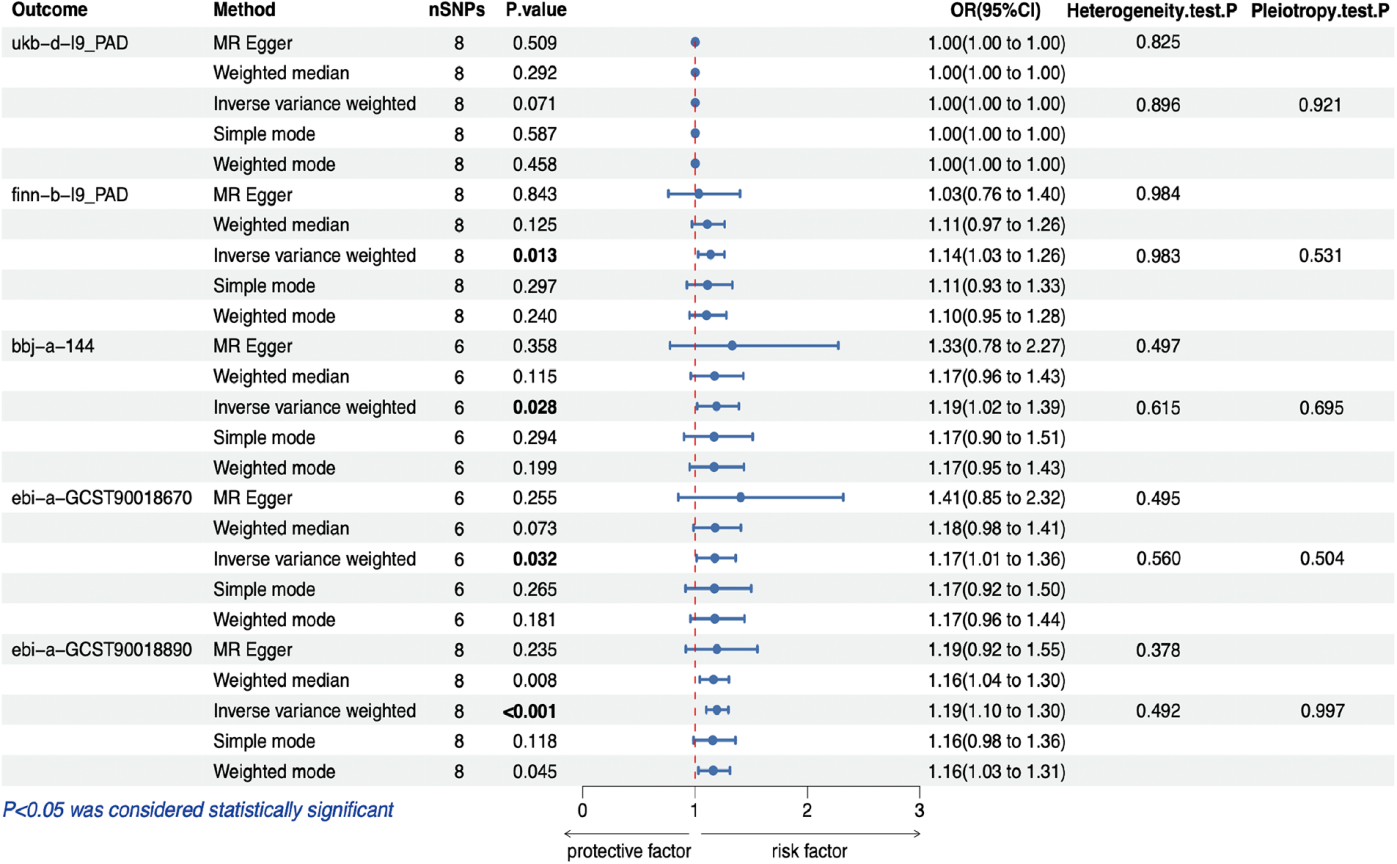
MR estimates for the relationship between PAD and obesity. MR, Mendelian randomization; PAD, peripheral artery disease; SNP, single nucleotide polymorphism; OR, odds ratios; CI, confidence interval.

Figure 2 illustrates the estimated causal effects of obesity on PAD. We observed a significant positive association between genetically predicted obesity and PAD in most of the datasets. IVW analysis revealed a significant positive relationship between genetic obesity and PAD (GWAS ID: finn-b-I9_PAD) (Odds Ratio (OR) = 1.138, 95% Confidence Interval (95% CI): 1.027–1.261, *p *= 0.013); a significant positive relationship between genetic obesity and PAD (GWAS ID: bbj-a-144) (OR = 1.190, 95% CI: 1.019–1.390, *p *= 0.028); a significant positive relationship between genetic obesity and PAD (GWAS ID: ebi-a-GCST90018670) (OR = 1.174, 95% CI: 1.014–1.360, *p *= 0.032); and a significant positive relationship between genetic obesity and PAD (GWAS ID: ebi-a-GCST90018890) (OR = 1.194, 95% CI: 1.099–1.296, *p *< 0.001). However, within the dataset (GWAS ID: ukb-d-I9_PAD), we did not observe a significant association between obesity and PAD (OR = 1.001, 95% CI: 1.000–1.002, *p *= 0.071). Furthermore, although the weighted median and MR-Egger analyses produced consistent estimates, they exhibited lower precision (Table 3). While we did not observe an increase in outcome heterogeneity and lacked evidence of directional pleiotropy (Table 4), we cannot completely eliminate the influence of both exposure and outcome data overlap due to the unavailability of the exact number of individuals with overlapping data.

**Table 3:** Mendelian randomization estimates for the relationship between PAD and obesity.

Exposure	Outcome	Method	nSNPs	β	SE	*P*	OR	95% CI	
								Lower	Upper
finn-b-E4_OBESITY	ukb-d-I9_PAD	MR Egger	8	8.27e-04	1.18e-03	0.509	1.001	0.999	1.003
finn-b-E4_OBESITY	ukb-d-I9_PAD	Weighted median	8	5.14e-04	4.88e-04	0.292	1.001	1.000	1.002
finn-b-E4_OBESITY	ukb-d-I9_PAD	IVW	8	7.12e-04	3.94e-04	0.071	1.001	1.000	1.002
finn-b-E4_OBESITY	ukb-d-I9_PAD	Simple mode	8	4.53e-04	7.96e-04	0.587	1.001	1.000	1.002
finn-b-E4_OBESITY	ukb-d-I9_PAD	Weighted mode	8	4.62e-04	5.88e-04	0.458	1.001	0.999	1.002
finn-b-E4_OBESITY	finn-b-I9_PAD	MR Egger	8	0.032	0.155	0.843	1.033	0.762	1.400
finn-b-E4_OBESITY	finn-b-I9_PAD	Weighted median	8	0.102	0.067	0.125	1.108	0.972	1.263
finn-b-E4_OBESITY	finn-b-I9_PAD	IVW	8	0.129	0.052	**0**.**013**	1.138	1.027	1.261
finn-b-E4_OBESITY	finn-b-I9_PAD	Simple mode	8	0.104	0.093	0.297	1.110	0.926	1.331
finn-b-E4_OBESITY	finn-b-I9_PAD	Weighted mode	8	0.097	0.076	0.240	1.102	0.950	1.278
finn-b-E4_OBESITY	bbj-a-144	MR Egger	6	0.284	0.274	0.358	1.329	0.777	2.273
finn-b-E4_OBESITY	bbj-a-144	Weighted median	6	0.159	0.101	0.115	1.172	0.962	1.429
finn-b-E4_OBESITY	bbj-a-144	IVW	6	0.174	0.079	**0**.**028**	1.190	1.019	1.390
finn-b-E4_OBESITY	bbj-a-144	Simple mode	6	0.155	0.132	0.294	1.168	0.901	1.512
finn-b-E4_OBESITY	bbj-a-144	Weighted mode	6	0.155	0.105	0.199	1.168	0.951	1.434
finn-b-E4_OBESITY	ebi-a-GCST90018670	MR Egger	6	0.340	0.256	0.255	1.405	0.851	2.318
finn-b-E4_OBESITY	ebi-a-GCST90018670	Weighted median	6	0.163	0.091	0.073	1.177	0.985	1.407
finn-b-E4_OBESITY	ebi-a-GCST90018670	IVW	6	0.161	0.075	**0**.**032**	1.174	1.014	1.360
finn-b-E4_OBESITY	ebi-a-GCST90018670	Simple mode	6	0.158	0.126	0.265	1.171	0.915	1.500
finn-b-E4_OBESITY	ebi-a-GCST90018670	Weighted mode	6	0.161	0.104	0.181	1.174	0.959	1.439
finn-b-E4_OBESITY	ebi-a-GCST90018890	MR Egger	8	0.178	0.135	0.235	1.194	0.917	1.555
finn-b-E4_OBESITY	ebi-a-GCST90018890	Weighted median	8	0.151	0.057	0.008	1.163	1.041	1.300
finn-b-E4_OBESITY	ebi-a-GCST90018890	IVW	8	0.177	0.042	**<0**.**001**	1.194	1.099	1.296
finn-b-E4_OBESITY	ebi-a-GCST90018890	Simple mode	8	0.146	0.082	0.118	1.157	0.985	1.358
finn-b-E4_OBESITY	ebi-a-GCST90018890	Weighted mode	8	0.149	0.061	0.045	1.161	1.029	1.309

PAD, peripheral artery disease; SNP, single nucleotide polymorphism; SE, standard error; OR, odd ratio; Cl, confidence interval; MR, Mendelian randomization; IVW, inverse variance weighted.

Values in bold indicate causal inference using the Inverse Variance Weighted (IVW) method, and the differences are statistically significant.

**Table 4:** Pleiotropy and heterogeneity test between peripheral artery disease and obesity.

Outcomes	Pleiotropy test			Heterogeneity test					
	MR-Egger			MR-Egger			Inverse-variance weighted		
	Intercept	SE	*p*	*Q*	*Q*_df	Q_pval	*Q*	*Q*_df	*Q*_pval
ukb-d-I9_PAD	−1.96e-05	1.89e-04	0.921	2.87	6	0.825	2.88	7	0.896
finn-b-I9_PAD	0.016	0.024	0.531	1.04	6	0.984	1.48	7	0.983
bbj-a-144	−0.017	0.041	0.695	3.38	4	0.497	3.56	5	0.615
ebi-a-GCST90018670	−0.028	0.038	0.504	3.39	4	0.495	3.93	5	0.560
ebi-a-GCST90018890	−9.33e-05	0.021	0.997	6.42	6	0.378	6.42	7	0.492

MR, Mendelian randomization; Q, heterogeneity statistic Q; df, degree of freedom.

### 3.3 Sensitivity analysis

In the sensitivity analysis of the causal relationship between obesity and PAD, we did not detect significant heterogeneity or horizontal pleiotropy. Cochran's *Q* test showed no heterogeneity (Table 4), and MR Egger regression displayed no horizontal pleiotropy (Table 4). Supplementary Material Figure S1 is a scatterplot illustrating individual estimates of the causal effect of obesity on PAD. It is evident that as the impact of SNPs on obesity becomes stronger, their effect on PAD also becomes more pronounced, indicating an overall positive association between genetic obesity and PAD. Supplementary Material Figure S2 presents a forest plot of SNPs associated with PAD risk, providing a more intuitive observation of the Beta values for each SNP after IVW calculation. These visual aids offer valuable insights into the consistency of our results. In addition to these figures, we conducted a leave-one-out sensitivity analysis, as shown in Supplementary Material Figure S3. This analysis method assesses the influence of individual SNPs on the overall estimate. Importantly, this analysis demonstrated the stability of the overall estimate, indicating that it was not unduly influenced by any specific SNP, and no evidence of reverse causality was found. Furthermore, we employed funnel plots to assess potential horizontal pleiotropy, as depicted in Supplementary Material Figure S4. The absence of asymmetry in this plot suggests no evidence of horizontal pleiotropy, further enhancing the reliability of our study results. Finally, we conducted a reverse causal MR analysis to explore the possibility of a reverse causal relationship between obesity and PAD. This comprehensive analysis revealed that there was no observed evidence of a reverse causal association between these two factors (Table 5). This finding further strengthens the notion that the relationship between obesity and PAD is primarily unidirectional, where obesity influences the risk of PAD rather than the other way around. This insight adds valuable depth to our understanding of the complex interplay between obesity and cardiovascular health.

**Table 5:** Reverse Mendelian randomization estimate of the relationship between PAD and obesity.

Outcome	Exposure	Method	nSNPs	*β*	SE	*P*	OR	95% CI	
								Lower	Upper
finn-b-E4_OBESITY	ukb-d-I9_PAD	IVW	2	37.369	29.995	0.213	1.70e + 16	4.98e-10	5.77e + 41
finn-b-E4_OBESITY	finn-b-I9_PAD	IVW	3	0.119	0.124	0.336	1.127	0.884	1.436
finn-b-E4_OBESITY	bbj-a-144	IVW	2	−0.038	0.143	0.789	0.962	0.727	1.274
finn-b-E4_OBESITY	ebi-a-GCST90018670	IVW	1	0.095	0.156	0.542	1.100	0.810	1.493
finn-b-E4_OBESITY	ebi-a-GCST90018890	IVW	5	0.107	0.090	0.231	1.113	0.934	1.327

PAD, peripheral artery disease; SNP, single nucleotide polymorphism; SE, standard error; OR, odd ratio; Cl, confidence interval; MR, Mendelian randomization; IVW, inverse variance weighted.

## 4 Discussion

In this study, we conducted a comprehensive investigation into the causal relationship between obesity and PAD using MR analysis. Our study results indicate that, after adjusting for smoking, hypertension, diabetes, and dyslipidemia, there is a genetic-level association between obesity and PAD in all the East Asian populations and most of the European populations included in the study. However, in one of the datasets derived from a European population, we did not detect a significant association. One plausible explanation for this lack of significance could be the influence of sample size (25), as we observed that within this dataset, the number of case groups is comparatively smaller while the number of control groups is larger than in other datasets.

Previous observational studies have uncovered a potential positive correlation between obesity and PAD. However, some studies have also raised the concept of the “obesity paradox” (6), which has spurred researchers to seek more evidence in order to establish a causal relationship between the two. An MR study conducted within the UK Biobank, involving 367,703 participants, has indicated that a higher BMI, particularly with a focus on the fat mass index, is associated with an elevated risk of several cardiovascular diseases, including PAD (26). Additionally, another MR study conducted in China, with a sample size of 11,447 adults, also suggested the presence of a causal link between obesity and PAD, even after accounting for potential intermediate factors like hypertension, dyslipidemia, and hyperglycemia (12). Our validation across multiple PAD datasets derived from GWAS corroborated the findings of the aforementioned studies, with the results remaining robust even after performing several sensitivity analyses aimed at eliminating potential pleiotropic instrumental variables. This provides further support for the potential causal relationship between obesity and PAD.

Common PAD risk factors include smoking, type 2 diabetes, high blood pressure, and high cholesterol (5). Obesity is closely related to physiological changes such as chronic inflammation and insulin resistance, and is the main cause of various metabolic diseases such as type 2 diabetes, fatty liver and hypertension (5). Previous studies have shown that in obese patients, adipose-related factors such as Retinol-Binding Protein-4 are significantly increased, which mediate the proliferation of vascular smooth muscle cells, and increase pro-inflammatory cytokines such as Tumor necrosis factor-alpha (TNF-alpha) and interleukins 1β, 2, 8, 10 (IL-1β, IL-2, IL-8, IL-10) and adhesion molecules such as vascular cell adhesion molecule 1, thereby aggravating the occurrence and development of PAD (5, 27). In addition, the excessive weight of obese people will increase the mechanical compression of the arteries of the lower limbs, leading to damage to the blood vessel wall and promoting the progression of PAD (28). Previous studies have shown that strategies aimed at reducing obesity reduce the risk of cardiovascular disease primarily by affecting downstream metabolic risk factors, particularly diabetes and hypertension (13). However, our study shows a potential association between obesity and PAD even after adjusting for variables such as diabetes, hypertension, smoking, and dyslipidemia. This seems to support obesity as an independent complex risk factor for PAD. This observation may guide our future research directions.

Furthermore, it's noteworthy that our study demonstrated a significant correlation between obesity and PAD in East Asian populations, although this association was not uniformly observed in all European populations. An earlier systematic study conducted among Hispanics found that despite a higher prevalence of risk factors such as hypertension, obesity, and diabetes when compared to non-Hispanic whites, Hispanics displayed notably lower rates of PAD and carotid artery disease, suggesting the existence of an “obesity paradox” within the Hispanic population (29). Simultaneously, multiple studies have suggested that individuals of African descent might exhibit a heightened susceptibility to PAD (1, 6, 30, 31). These findings imply the presence of racial disparities in the causal relationship between obesity and PAD, emphasizing the significance of studying diverse ethnic groups in our future research.

Nevertheless, this study does have some limitations that warrant consideration. Firstly, while the MR-Egger intercept test did not reveal evidence of pleiotropic effects, it's crucial to acknowledge that completely ruling out the potential for directional pleiotropy in any MR study remains challenging (11). Secondly, our instrumental variables primarily derive from European populations, while our study encompasses East Asian populations. Therefore, we cannot overlook the potential impact of racial disparities on the results, thus affecting the generalizability of our findings. Additionally, although we recognize the significant relevance of the Rutherford classification to the treatment and prognosis of PAD at different stages (32), the limitations of our dataset precluded further subgroup analysis. Consequently, the findings of this study are subject to certain constraints. Moving forward, we aim to enhance and validate our research outcomes by refining the Rutherford classification information and conducting prospective studies involving large, racially diverse samples. Thirdly, the relatively limited number of SNPs we were able to obtain may not fully eliminate bias, primarily due to the restricted sample size. Finally, it's imperative to recognize that our assessment of causality was based on MR, which utilizes the genetic information of each trait, necessitating cautious interpretation, as both obesity and the development of PAD are multifactorial in nature.

## 5 Conclusions

In summary, our study provides genetic evidence suggesting a potential causal relationship between obesity and PAD. Although we did not find evidence supporting the “obesity paradox”, careful weight management is still important, as lower weight does not necessarily lead to better outcomes. As with any study, caution is needed in interpreting the findings. Further research is necessary to investigate the clinical relevance of weight in preventing PAD, as this could aid in developing more precise intervention strategies.

## Data Availability

The original contributions presented in the study are included in the article/**Supplementary Material**, further inquiries can be directed to the corresponding authors.
