# Supplementary figures and images for: Assessment of causal associations between obesity and peripheral artery disease: a bidirectional Mendelian randomization study
Source: Front Cardiovasc Med. 2024 May 6;11:1332530. doi: 10.3389/fcvm.2024.1332530 (PMC11107290; doi:10.3389/fcvm.2024.1332530)

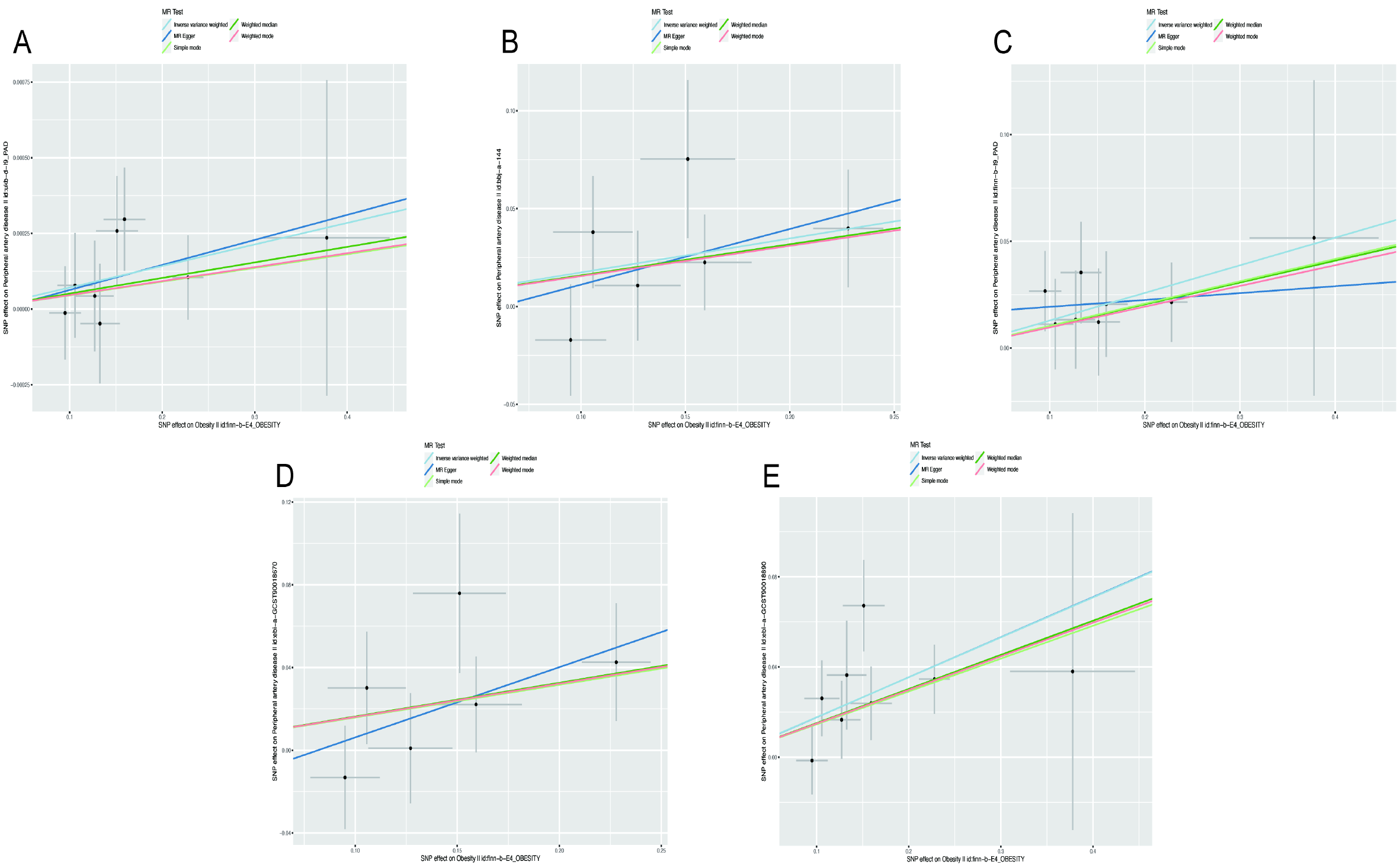

Supplement: Supplementary file 1 [file Image1.tif]

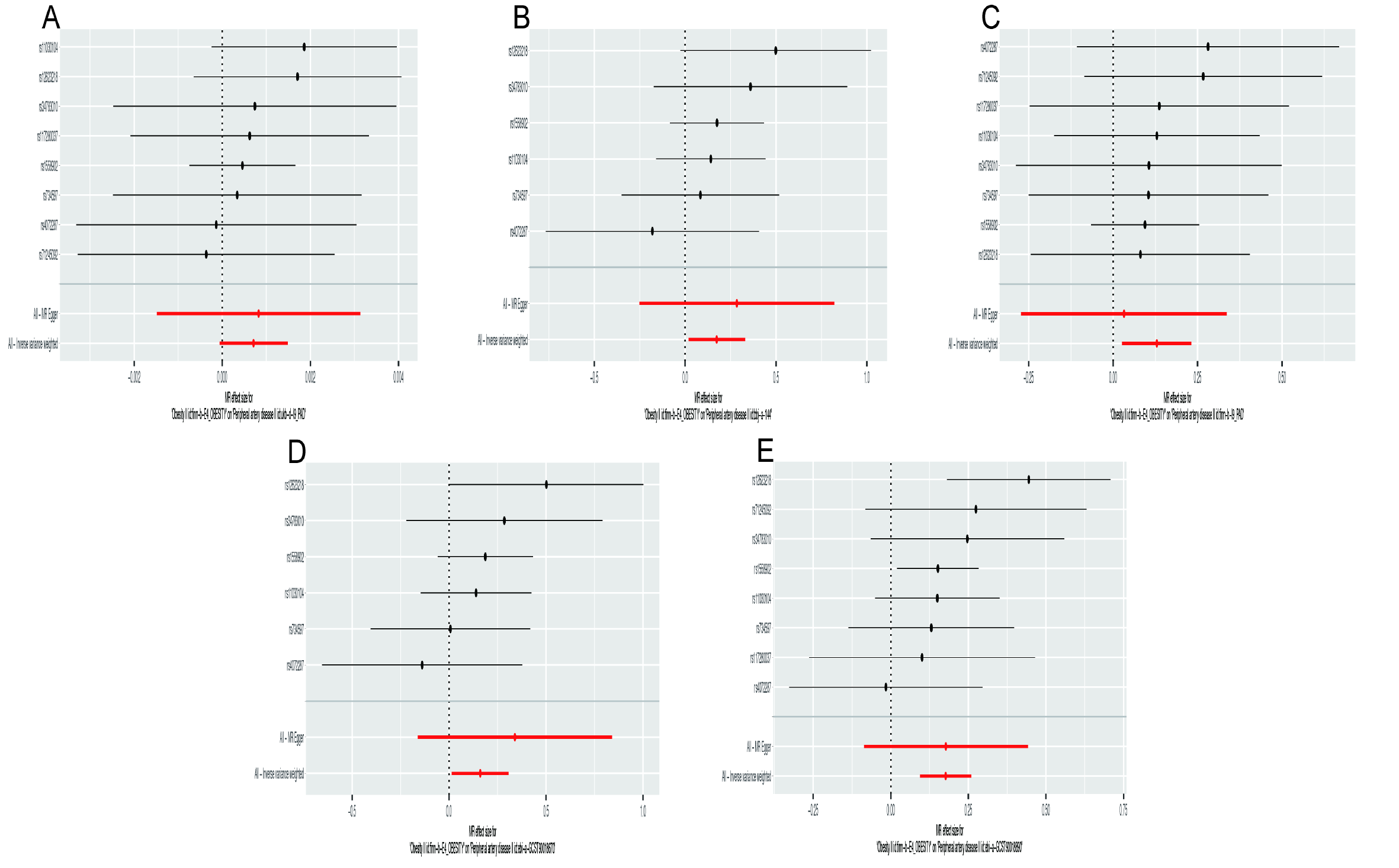

Supplement: Supplementary file 2 [file Image2.tif]

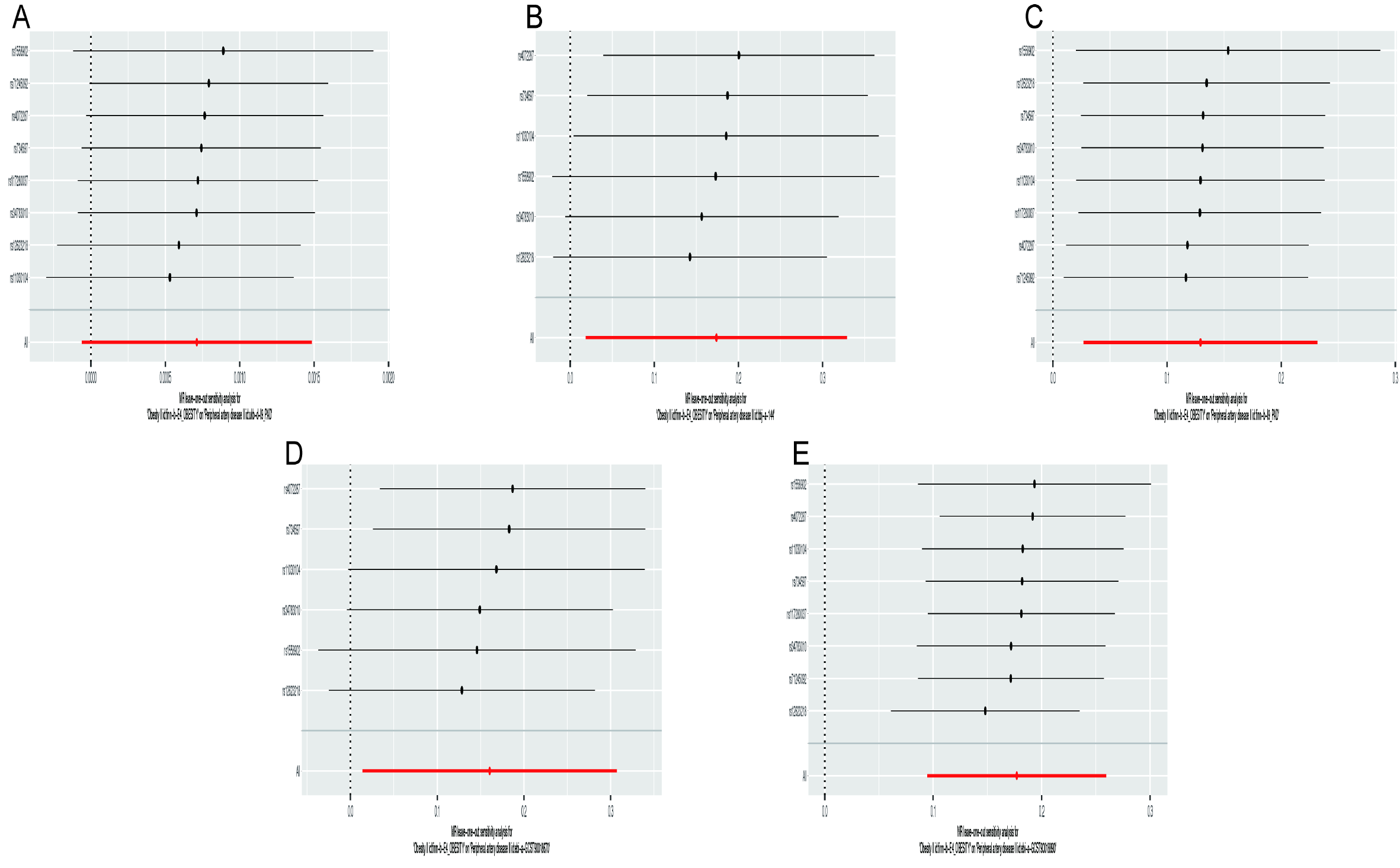

Supplement: Supplementary file 3 [file Image3.tif]

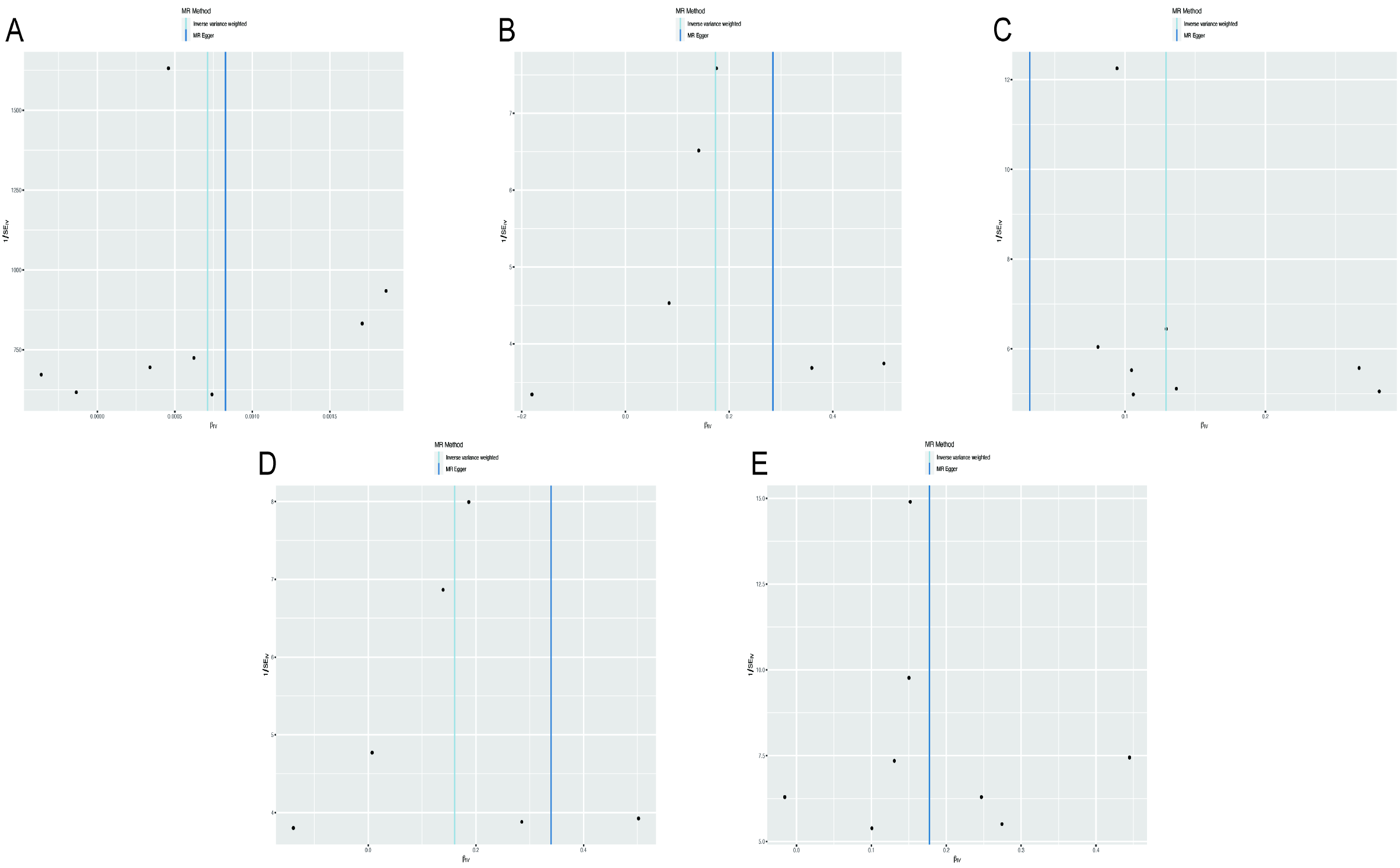

Supplement: Supplementary file 4 [file Image4.tif]
